# Effects of acupuncture and nicotine patch on smoking: a multicenter, randomized, controlled, double-blind clinical trial

**DOI:** 10.3389/fmed.2024.1418967

**Published:** 2024-07-17

**Authors:** Runjing Dai, Dong Ren, Binning Li, Yanfeng Zhang, Xiaojing Ma, Xiangrong Zhang, Hailiang Zhang, Lina Zhang, Chenchen Zeng, Xiaomei Jiang, Shisan Bao, Jingchun Fan

**Affiliations:** ^1^Hospital Infection-Control Department, Xi‘an Aerospace General Hospital, Xi’an, Shaanxi, China; ^2^School of Public Health, Center for Evidence-Based Medicine, Gansu University of Chinese Medicine, Lanzhou, Gansu, China; ^3^Department of Psychosomatic and Sleep Medicine, Gansu Gem Flower Hospital, Lanzhou, Gansu, China; ^4^Library, Gansu University of Chinese Medicine, Lanzhou, Gansu, China; ^5^School of Acupuncture and Tuina, Gansu University of Chinese Medicine, Lanzhou, Gansu, China; ^6^Department of Chinese Medicine, Health Center of Hekou Town, Lanzhou, Gansu, China

**Keywords:** acupuncture, nicotine patch, combined treatment, smoking cessation, withdraw syndrome, randomized clinical trial

## Abstract

**Aims:**

To evaluate the effects of acupuncture and/or nicotine patches on smoking cessation.

**Methods:**

Eighty-eight participants were randomly allocated into four groups: acupuncture combined with nicotine patch (ACNP), acupuncture combined with sham nicotine patch (ACSNP), sham acupuncture combined with nicotine patch (SACNP), and sham acupuncture combined with sham nicotine patch (SACSNP). The primary outcome was self-reported smoking abstinence verified with expiratory Carbon Monoxide (CO) after 8 weeks of treatment. The modified Fagerstrom Test for Nicotine Dependence (FTND) score, Minnesota Nicotine Withdrawal Scale (MNWS), and the Brief Questionnaire of Smoking Urge (QSU-Brief) score were used as secondary indicators. SPSS 26.0 and Prism 9 software were used for statistical analyses.

**Results:**

Seventy-eight participants completed the study. There were no significant differences in patient characteristics at baseline across the four groups. At the end of treatment, there was a statistically significant difference (*χ^2^* = 8.492, *p* = 0.037) in abstaining rates among the four groups. However, there were no significant differences in the reduction in the number of cigarettes smoked daily (*p* = 0.111), expiratory CO (*p* = 0.071), FTND score (*p* = 0.313), and MNWS score (*p* = 0.088) among the four groups. There was a statistically significant difference in QUS-Brief score changes among the four groups (*p* = 0.005). There was no statistically significant interaction between acupuncture and nicotine patch.

**Conclusion:**

Acupuncture combined with nicotine replacement patch therapy was more effective for smoking cessation than acupuncture alone or nicotine replacement patch alone. No adverse reactions were found in the acupuncture treatment process.

**Clinical trial registration:**

http://www.chictr.org.cn/showproj.aspx?proj=61969, identifier ChiCTR2100042912.

## Introduction

Smoking, recognized as one of the most serious global public health issues, is a significant risk factor for life-threatening diseases such as lung cancer, coronary heart disease, and stroke (1, 2). According to the World Health Organization, the tobacco industry exacts a toll of more than 8 million human lives, 600 million trees, 200,000 hectares of land, 22 billion tons of water, and 84 million tons of CO_2_ annually (3). Cigarette smoke contains over 5,000 harmful chemicals, with 69 identified as carcinogenic (4). Apart from its health impact, smoking imposes a considerable economic burden, particularly in low- and middle-income countries (5, 6), reducing tobacco consumption and promoting smoking cessation through various interventions, including socioeconomic, non-pharmaceutical, and pharmaceutical approaches, is crucial (7).

Nicotine replacement therapy (NRT) is a widely used pharmaceutical intervention for smoking cessation (8). NRT, available as Over-the-Counter (OTC) drugs (9), includes patches, gum, nasal sprays, and inhalers (10, 11).

Recently, acupuncture for smoking cessation has gained attention globally (12). Studies, such as a large-scale observational study in Hong Kong and a randomized controlled trial, have suggested that acupuncture is a safe and effective method for smoking cessation (13, 14). Meta-analyses have shown its safety but called for further trials to establish effectiveness (15, 16). Acupuncture offers advantages, including mood improvement and countering appetite loss associated with NRT (17). When combined with counseling or educational programs, acupuncture enhances its efficacy for sustained smoking cessation compared to standalone interventions (18). Despite these findings, variations in study designs necessitate further exploration to comprehensively validate acupuncture’s effectiveness in smoking cessation.

To address this, we conducted a randomized controlled double-blind trial to investigate the effectiveness and safety of acupuncture and/or nicotine patches with a placebo in facilitating smoking cessation, aiming to enhance smoking cessation programs with more effective methods and reduced relapse rates.

## Methods

### Study design

This study employed a randomized, controlled, double-blind, double-dummy, multicenter trial to assess the effects and safety of acupuncture (or sham acupuncture) combined with nicotine patch (or placebo patch) for smoking cessation. The study, registered in the Chinese Clinical Trial Registry with the registration number ChiCTR2100042912 on January 31, 2021.^1^

### Participant recruitment

We recruited 88 smokers from three districts in Lanzhou City, Gansu Province, China (Chengguan, Xigu, and Heping). Recruitment involved posters and the popular Chinese social media platform, WeChat, to connect with ethnic communities and universities for community smoking cessation programs. All participants voluntarily participated in the study and provided written informed consent.

#### Inclusion criteria

Participants were required to meet the following conditions:

1. Meet diagnostic criteria for smoking, including daily tobacco product use for at least 3 weeks and experiencing withdrawal symptoms within 24 h of cessation or reduction.
2. Voluntarily quit smoking.
3. Aged ≥16 years.
4. Signed informed consent.

#### Exclusion criteria

1. Participants with severe heart, brain, lung-related system diseases, and diabetes were excluded, along with those who received other relevant smoking cessation treatment in the past 2 weeks.
2. Individuals allergic to the nicotine patch adhesive or with conditions elevating the risk of bleeding or taking medications increasing the risk of bleeding were also excluded.

### Sample size

The sample size was calculated based on a completely random design, assuming abstinence rates of 56% (p1) (19) and 9.1% (p2) (20) for acupuncture and placebo, respectively. The required sample size for each group was 22, considering a 20% loss to follow-up rate.

The equation of sample size calculation is *N* $=\frac{\left[Z_{1-\alpha /2}\sqrt{2\bar{p}\left(1-\bar{p}\right)}\pm Z_{\beta }\sqrt{p_{1}\left(1-p_{1}\right)+p_{2}\left(1-p_{2}\right)}\right]2}{\left(p_{1}-p_{2}\right)2}$ .

### Randomized allocation of participants

We generated a set of continuous serial numbers from 1 to 88 prior to enrollment. Participants corresponded to the corresponding serial numbers according to the order of enrollment. Excel was used to generate 88 random numbers, and the random numbers were numbered in the order from smallest to largest. If the same random numbers appeared, the first appeared was small. Participants were randomly assigned in a 1:1:1:1 ratio to four intervention groups: acupuncture combined with nicotine patch (ACNP), acupuncture combined with sham nicotine patch (ACSNP), sham acupuncture combined with nicotine patch (SACNP), and sham acupuncture combined with sham nicotine patch (SACSNP).

### Blinding

This trial adopted a randomized, controlled, double-blind, double-dummy, multicenter experimental design. Participants and data collectors were blinded. The randomization sequence was inserted into sequentially numbered, opaque, sealed envelopes. The acupuncturist treating the patient opened these envelopes (21).

### Interventions

**Figure 1:**
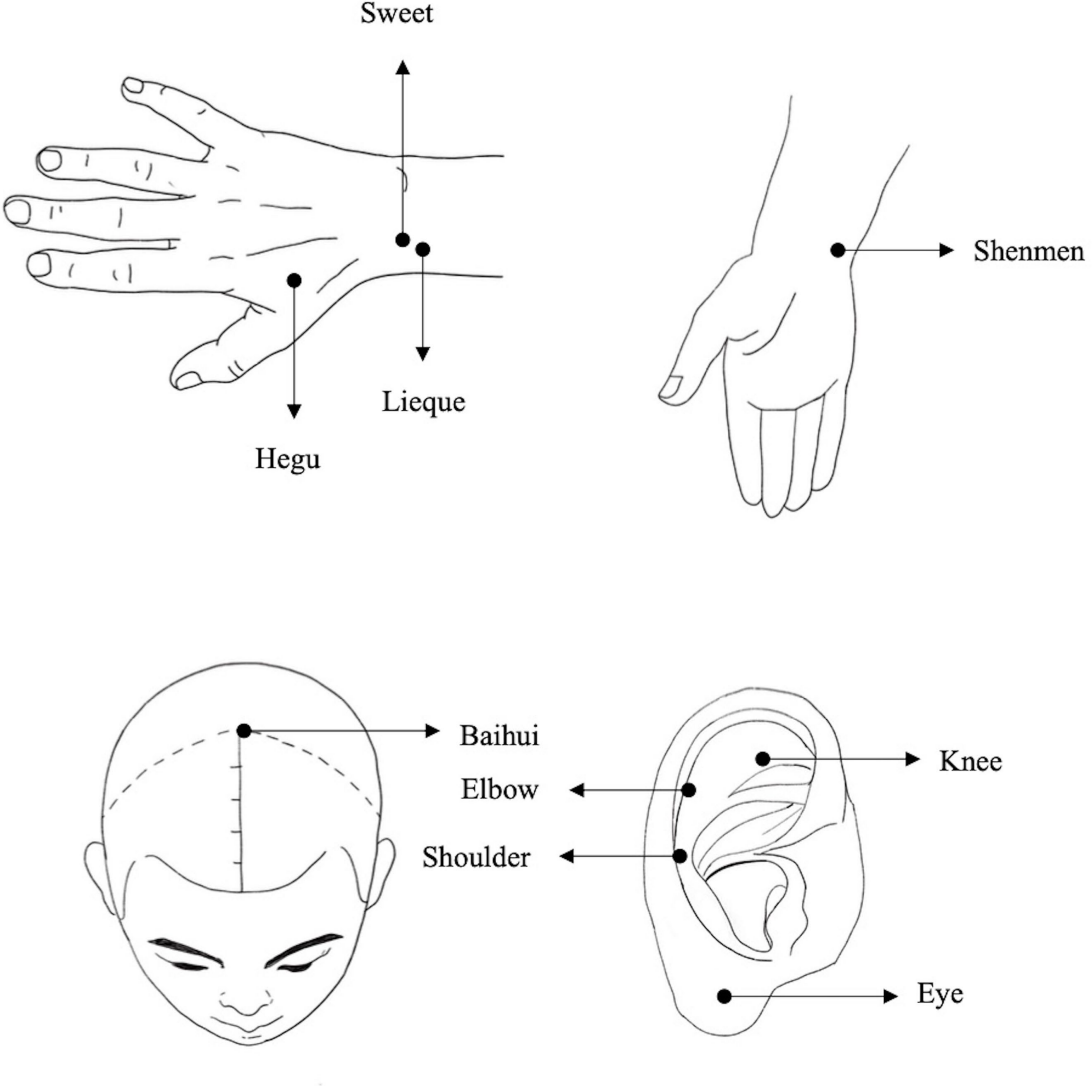
Acupuncture and sham acupuncture points map.

Each participant received acupuncture (or sham acupuncture) and nicotine patch (or placebo patch) for 8 weeks, followed by an 8-week follow-up. Following extensive literature search, the follow-up period varied greatly, ranging from 2 weeks to 1 year. Considering the compliance of the participants, 8 weeks of follow-up chosen was almost the best. The acupuncture points selected for smoking cessation in the current study are based on previous published researches (22, 23). In more details, Baihui acupoint on the head has the function of awakening the brain (24), Lieque point belongs to lung meridian, can treat lung diseases (25). Hegu and Shenmen points can harmonize qi and blood, tranquilize and calm the mind (26, 27), and Sweet point is a special point to reduce smoking addiction (28). It has been well accepted by the Traditional Chinese Medicine practitioners that multiple points combination therapy offers more effective approaches than single point treatment. Thus, these five combined points acupunctures have been selected for smoke cessation in the current study. Sham acupuncture was selected as a component of the control. The sham acupuncture targeted corresponding shoulder, eye, knee, and elbow acupoints on the auricle that are unrelated to smoking cessation (29) (Figure 1). Each participant received three sessions of treatment per week, while we established a WeChat (social media) group for patients to remind each participant of the appointment time and improve treatment compliance.

The nicotine patch dose was based on daily smoking and body weight (Table 1). Nicotine patch refers to a patch that absorbs nicotine through the skin (transdermal patch); while the sham patches were the patch without nicotine.

**Table 1:** Duration and dosage of nicotine patch.

Daily smoking/weight	Week 1–2	Week 3–4	Week 5–8
≥20	2 patches/day	1 patch/day	0.5 patches/day
<20/ ≥70 kg	2 patches/day		
<20/<70 kg	1 patch/day		

The amount of nicotine patch used should be determined by the amount of cigarettes smoked each day. For participants who smoke more, the cycle of 2 patches/ day should be appropriately extended.

### Adverse events

Adverse events in this study included needle fainting, stagnation, bending, folding and allergy during the use of the patch (21), as well as, adverse reactions such as bleeding, hematoma, dizziness, fainting, residual needle sensation, tenderness and infection during the treatment. Adverse events were graded according to the severity of the symptoms/signs using a 3-point scale, 1 = mild (easily tolerated, causing minimal discomfort), 2 = moderate (discomfort significant enough to interfere with daily activities) and 3 = severe (incapacitating and/or requiring therapeutic intervention). The relationship between adverse events and interventions will be rated by participants as follows: 1 = not relevant, 2 = possibly, 3 = probably, 4 = definitely, and were monitored and records (30). Participants experiencing adverse reactions were assessed by the attending physician, who decided on trial continuation or cessation.

### Outcome measures

Baseline characteristics were collected, and the primary outcome was the abstinence rate at the end of treatment, verified by expiratory Carbon Monoxide (CO < 10 ppm) (31) and/or self/report validation. To reduce/minimize the possible bias, we conducted multiple self-reports over the study, as described (21). At the meantime, we combined the observation reports of the participants’ families and other medical personnel to obtain more comprehensive information.

Secondary outcomes included measures of nicotine dependence (Fagerstrom test for nicotine dependence, FTND score), withdrawal symptoms (Minnesota Nicotine Withdrawal Scale, MNWS score), desire to smoke (The brief questionnaire of smoking urge, QSU-Brief score), expiratory CO value (measured by CO detector) and blood pressure (measured by Electronic Blood Pressure Monitor).

### Statistical methods

Intent-to-treat (ITT) and per-protocol (PP) analyses were conducted using SPSS 26.0 and Prism 9 software. Statistical significance was set at *p* ≤ 0.05.

### Ethical aspects/considerations

The study adheres to the Helsinki Declaration, approved by the Human Ethics Committee of the Affiliated Hospital of Gansu University of Chinese Medicine (Approval No. 20203), and registered at the Chinese Clinical Trial Registry (Registration Number ChiCTR2100042912).

## Results

### Recruitment and dropout summary

**Figure 2:**
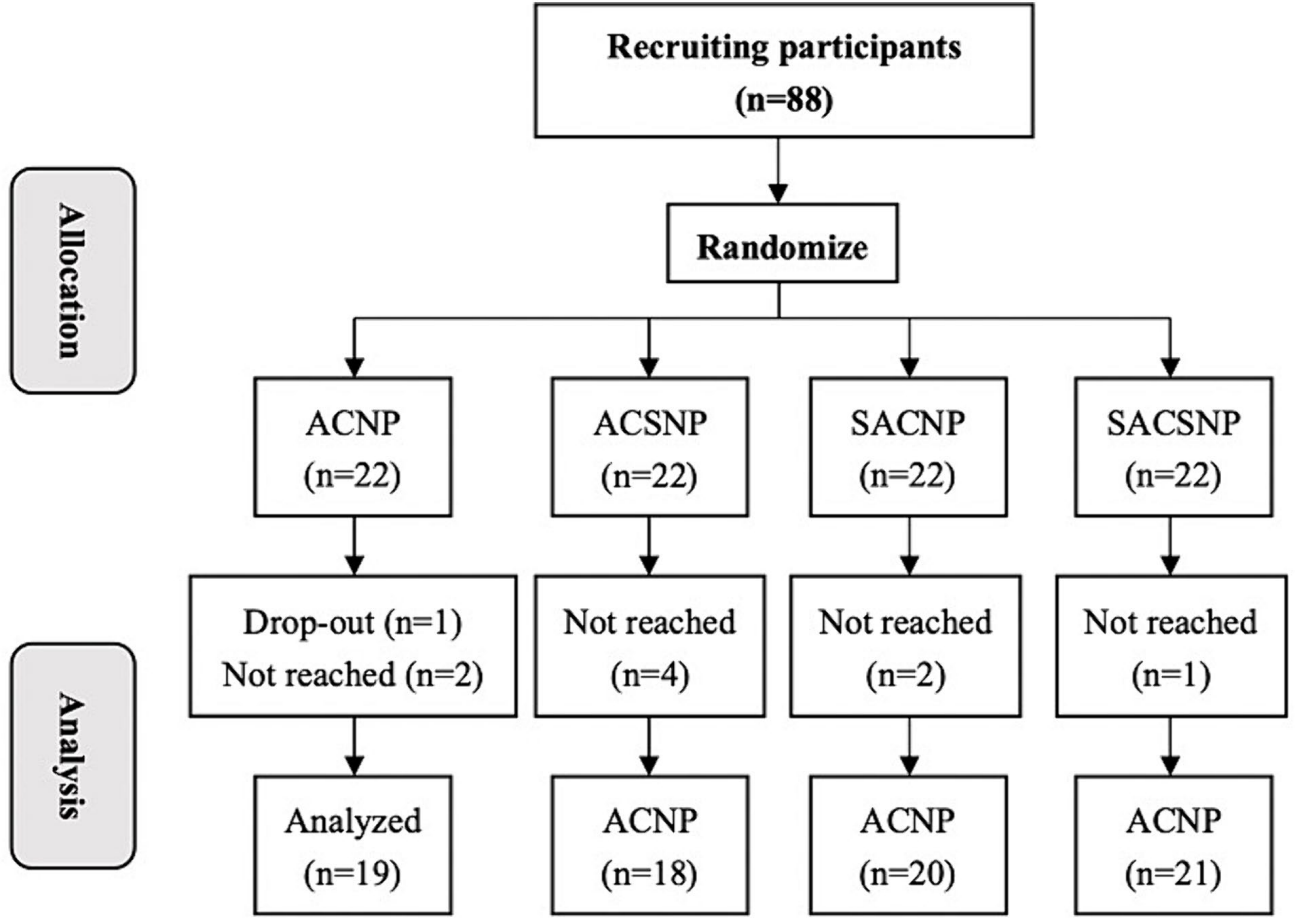
Subject intervention flow chart.

We enrolled a total of 88 eligible participants from Chengguan, Xigu, and Heping Districts, Lanzhou City, Gansu Province, China, between August 2021 and November 2021. These participants were then assigned to 4 treatment groups. Out of them, 78 participants completed the treatment, and 10 dropped out. Specifically, there were 3 dropouts in the ACNP group, 4 in the ACSNP group, 2 in the SACNP group, and 1 in the SACSNP group. Notably, only 1 participant in the ACNP group voluntarily withdrew due to job changes, while the remaining 9 participants discontinued treatment because of the impact of the COVID-19 epidemic (Figure 2).

### Participant characteristics at baseline

No significant differences in participant characteristics were observed across groups, including sex, age, demographic variables, BMI, smoking age, FTND score, MNWS score, and QSU-Brief score, as presented in Table 2. Statistical analysis of baseline data on daily cigarette consumption also indicated a balanced distribution among smokers across all groups at the trial’s outset (Table 2).

**Table 2:** Baseline characteristics comparison among all groups.

Characteristic	ACNP (*n* = 22)	ACSNP (*n* = 22)	SACNP (*n* = 22)	SACSNP (*n* = 22)	*F/Z/χ^2^*	*p*
Demographic information						
Age (ys)	40.45 ± 2.82	44.27 ± 2.52	42.09 ± 3.34	46.32 ± 3.17	0.738	0.532
Sex, *n* (%)						
Male	21 (95.5)	22 (100)	21 (95.5)	22 (100)	2.157	1.000
Female	1 (4.5)	0 (0)	1 (4.5)	0 (0)		
BMI [kg/m2, M (P25, P75)]	25.08 (23.01, 29.04)	24.61 (22.47, 26.39)	23.73 (21.87, 28.39)	24.15 (23.08, 25.51)	2.013	0.570
Education, *n* (%)						
Primary school Middle school	1 (4.5)	0 (0)	1 (4.5)	2 (9.1)	15.432	0.172
Middle school	5 (22.7)	5 (22.7)	4 (18.2)	5 (22.7)		
High school	1 (4.5)	2 (9.1)	8 (36.4)	6 (27.3)		
College degree	5 (22.7)	7 (31.8)	3 (13.6)	6 (27.3)		
≥ Bachelor degree	10 (45.6)	8 (36.4)	6 (26.3)	3 (13.6)		
Marital status, *n* (%)						
Unmarried	6 (27.3)	2 (9.1)	7 (31.8)	4 (18.2)	5.266	0.455
Married	15 (68.2)	19 (86.4)	15 (68.2)	17 (77.3)		
Divorced	1 (4.5)	1 (4.5)	0 (0)	1 (4.5)		
Smoking-related variables						
Smoking years	21.41 ± 2.14	25.45 ± 2.78	24.18 ± 2.98	26.64 ± 2.37	0.753	0.523
Cigarettes daily smoked M (P25, P75)	20.00 (12.25, 27.75)	20.00 (15.00, 30.00)	20.00 (15.00, 27.75)	18.00 (12.75, 20.00)	0.844	0.839
Expiratory CO M (P25, P75)	12.25 (10.17, 13.98)	10.90 (8.15, 13.53)	11.35 (6.50, 16.10)	11.30 (8.17, 15.33)	1.178	0.758
FTND score M (P25, P75)	6.00 (3.75, 8.00)	5.00 (3.00, 7.25)	7.00 (5.50, 7.00)	5.00 (3.75, 7.00)	2.720	0.437
MNWS score	25.36 ± 2.24	22.27 ± 2.53	25.14 ± 2.27	20.95 ± 2.36	1.131	0.341
QSU-Brief score	39.50 ± 3.19	39.95 ± 3.04	38.18 ± 3.07	32.55 ± 2.30	1.368	0.258

### Tobacco abstinence rate at the end of treatment

**Figure 3:**
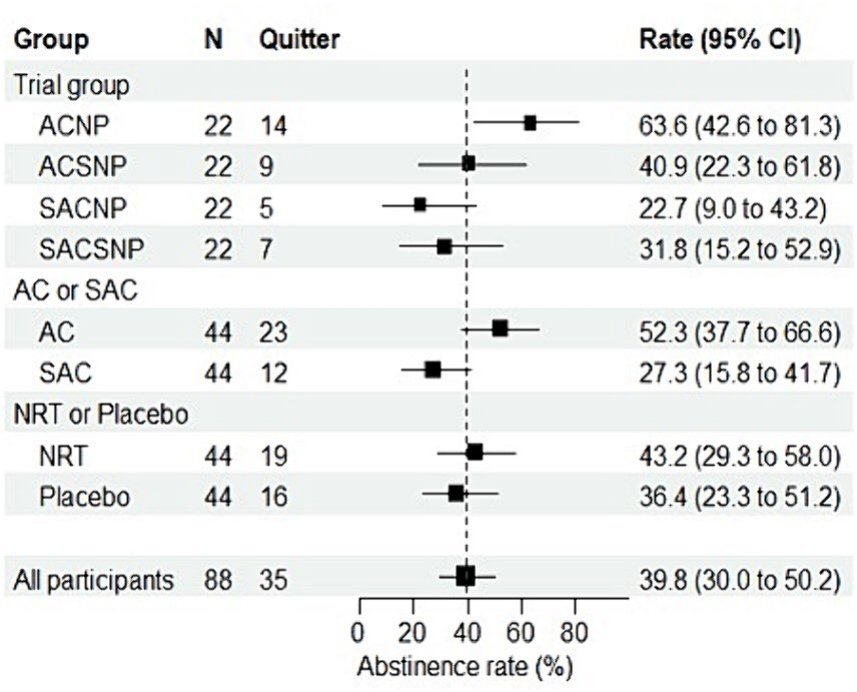
Forest plot of ITT-based abstinence rate (%) by trial groups at the end of treatment.

The self-reported overall abstinence rate stood at 39.8% (*95% CI*: 30.0 to 50.2%). According to the ITT analysis, the self-reported abstaining rates were 63.6, 40.9, 22.7, and 31.8%, respectively, in the ACNP, ACSNP, SACNP, and SACSNP groups, with a statistically significant difference (*χ^2^* = 8.492, *p* = 0.037). Figure 3 illustrates that the self-reported abstinence rate in the ACNP group surpassed the other groups, with pairwise comparisons revealing significantly higher rates compared to the SACNP group (*χ^2^* = 7.503, *p* = 0.006) and the SACSNP group (*χ^2^* = 4.464, *p* = 0.035). The per-protocol analysis confirmed significant differences among groups (*χ^2^* = 10.891, *p* = 0.012), with pairwise comparisons showing distinctions between the ACNP group and the SACNP group (*χ^2^* = 9.244, *p* = 0.004), and between the ACNP group and the SACSNP group (*χ^2^* = 6.513, *p* = 0.014) (Table 3). ITT analysis based on self-report and expiratory CO demonstrated significant differences among all groups (*χ^2^* = 10.907, *p* = 0.012), with a statistically significant difference in PP analysis as well (*χ^2^* = 13.187, *p* = 0.004).

**Table 3:** Comparison of tobacco abstinence rate among groups at the end of treatment.

Standard of judgment	Methods	Group	*n*	Give up smoking	Not give up smoking	Abstinence/relapse rate	*χ^2^*	*p*
Based on the self-reported	Intention-to-treat (ITT) analysis	ACNP	22	14	8	63.6^*	8.492	0.037
		ACSNP	22	9	13	40.9		
		SACNP	22	5	17	22.7		
		SACSNP	22	7	15	31.8		
	Per-protocol (PP) analysis	ACNP	19	14	5	73.7^*	10.891	0.012
		ACSNP	18	9	9	50.0		
		SACNP	20	5	15	25.0		
		SACSNP	21	7	14	33.3		
Based on the self-reported and expiratory CO	Intention-to-treat (ITT) analysis	ACNP	22	14	8	63.6^*	10.907	0.012
		ACSNP	22	7	15	31.8		
		SACNP	22	5	17	22.7		
		SACSNP	22	5	17	22.7		
	Per-protocol (PP) analysis	ACNP	19	14	5	73.7^*	13.187	0.004
		ACSNP	18	7	11	38.9		
		SACNP	20	5	15	25.0		
		SACSNP	21	5	16	23.8		

*p*^ ≤ 0.05 compared with the SACNP group; p* ≤0.05 compared with the SACSNP group.

Regardless of NRT or placebo treatment, participants receiving acupuncture exhibited a significantly higher abstinence rate than those undergoing sham acupuncture. However, participants receiving NRT only slightly outperformed those receiving sham NRT (Figure 3).

### The change of the number of cigarettes daily smoked at the end of treatment

**Figure 4:**
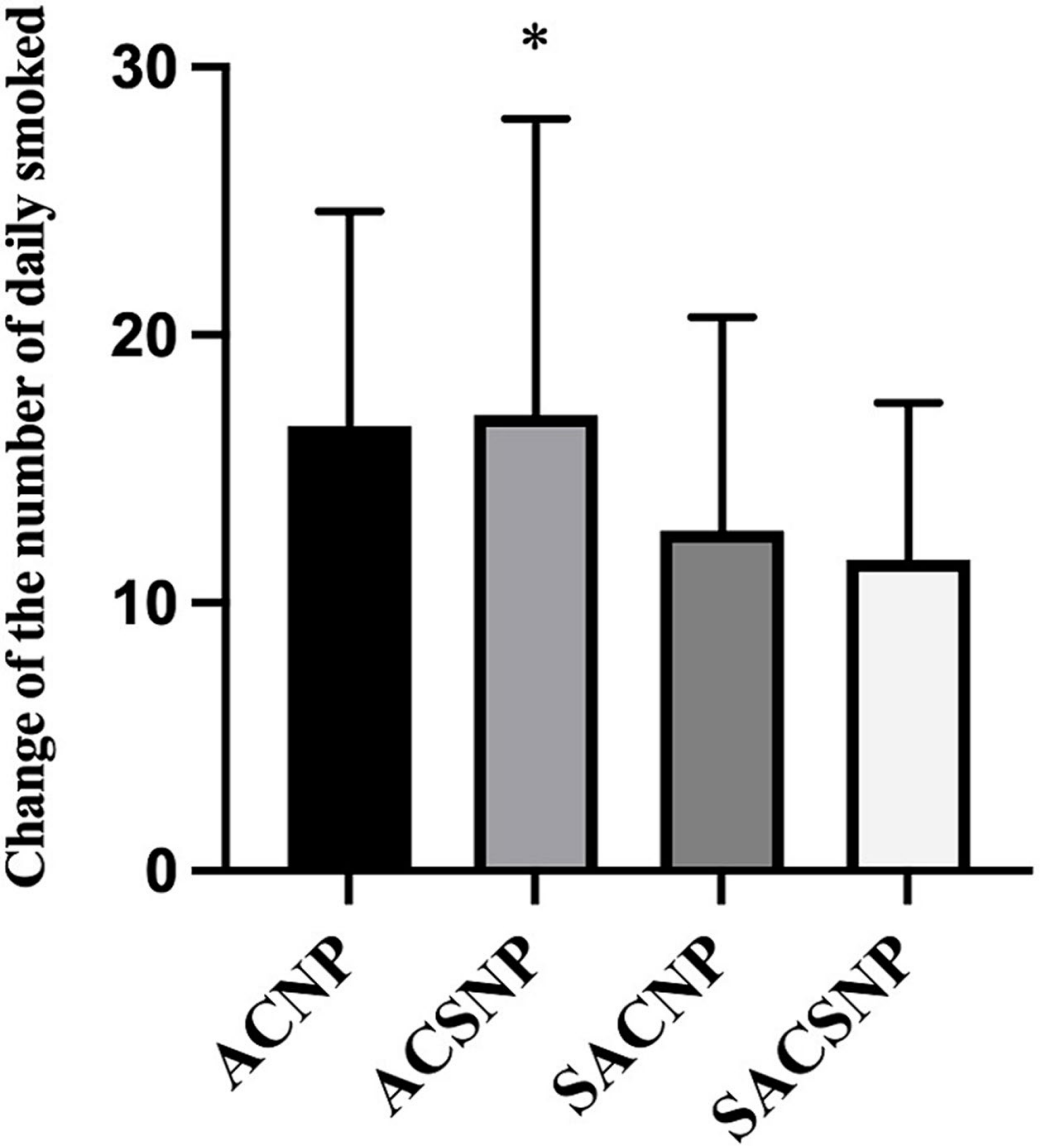
The reduction in the number of daily smoked.

While there was no statistical difference in the change of the number of cigarettes smoked daily across the groups (*F* = 2.075, *p* = 0.111), pairwise comparisons revealed that the ACSNP group exhibited a higher reduction in daily cigarettes smoked compared to the SACSNP group (*p* = 0.048) (Figure 4).

### The reduction in expiratory CO, FTND score, MNWS score and QUS score

**Figure 5:**
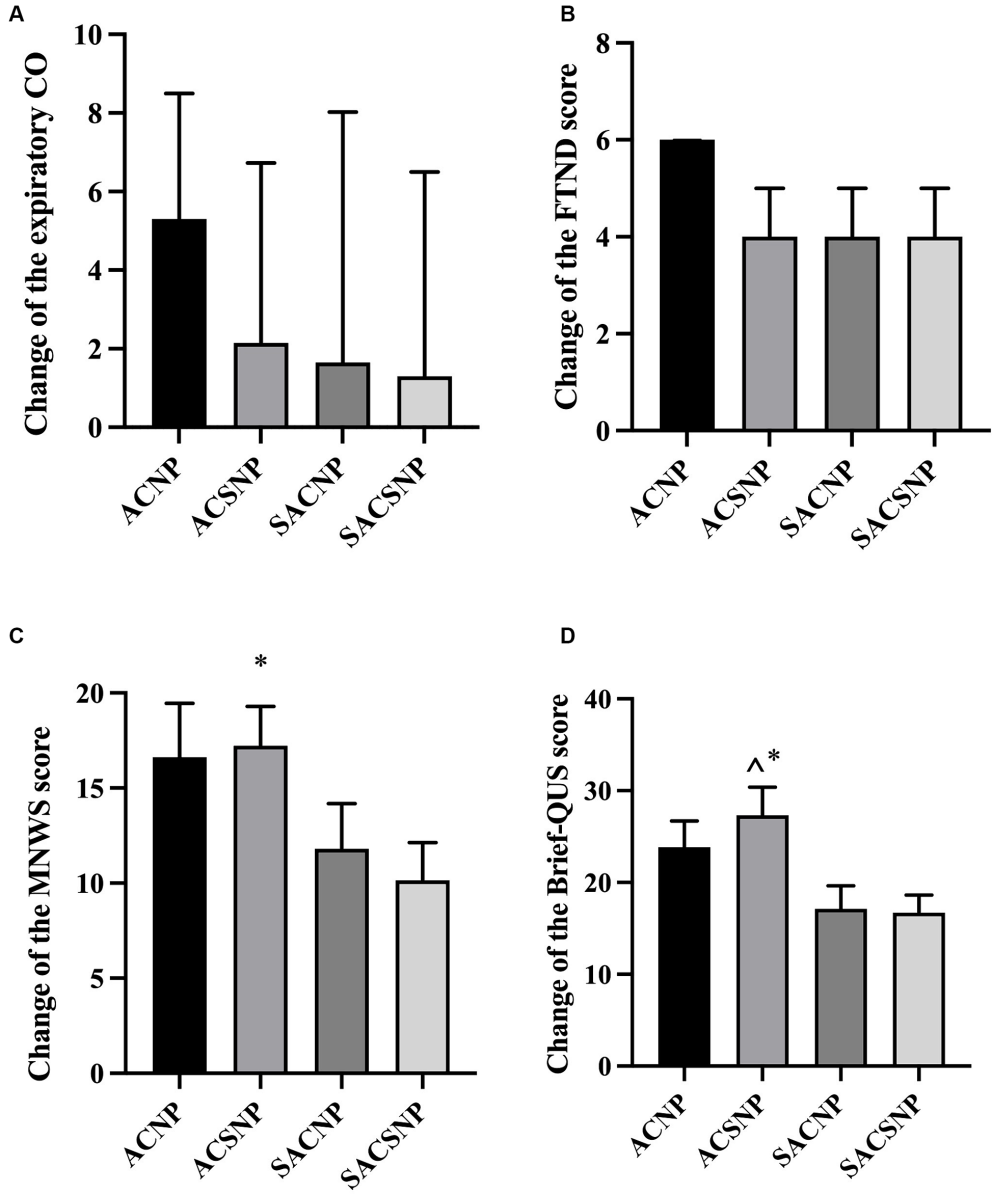
Changes of outcome indexes before and after treatment. **(A)** Change of the expiratory CO before and after treatment. **(B)** Change of the FTND score before and after treatment. **(C)** Change of the MNWS score before and after treatment. **(D)** Change of the Biref-QUS score before and after treatment.

There was no significant difference in the change of expiratory CO (*Z* = 7.015, *p* = 0.071) (Figure 5A), FTND score (*Z* = 3.558, *p* = 0.313) (Figure 5B), and the MNWS score (*F* = 2.265, *p* = 0.088) (Figure 5C) across the groups from baseline to the end of treatment. However, in pairwise comparisons, the ACSNP group was found to be more effective in reducing MNWS score than the SACSNP group (*p* = 0.036). Additionally, there was a statistical difference in the change of QUS-Brief score across the four groups (*F* = 4.013, *p* = 0.011), and the pairwise comparison results indicated that the ACSNP had a better effect than the SACNP (*p* = 0.007) and SACSNP (*p* = 0.005) (Figure 5D).

### Interaction analysis of acupuncture and nicotine patch on expiratory CO, change in the number of cigarettes smoked daily

The results of the interaction analysis revealed no significant difference between acupuncture and sham acupuncture in reducing expiratory CO content (*F* = 1.629, *p* = 0.206). However, acupuncture showed a more pronounced effect on the change in the number of cigarettes smoked daily (*F* = 6.005, *p* = 0.017). The nicotine patch was more effective in reducing expiratory CO than the sham nicotine patch (*F* = 4.565, *p* = 0.036). No significant interaction was observed between acupuncture and the nicotine patch in reducing expiratory CO content (*F* = 0.136, *p* = 0.687) or the number of cigarettes smoked daily (*F* = 0.158, *p* = 0.692) (Table 4).

**Table 4:** The interaction analysis of acupuncture and nicotine patch on expiratory CO and the change in the number of cigarettes smoked daily.

Effect	Cause	*F*	*p*
Acupuncture	Expiratory CO	1.629	0.206
	Change in the number of cigarettes smoked daily	6.005	0.017
Nicotine patch	Expiratory CO	4.565	0.036
	Change in the number of cigarettes smoked daily	0.030	0.862
Acupuncture* Nicotine patch	Expiratory CO	0.163	0.687
	Change in the number of cigarettes smoked daily	0.158	0.692

### Tobacco relapse rate

Among participants who had successfully quit smoking by the end of the treatment, 2 individuals in the ACSNP group resumed smoking during the follow-up, while no relapses occurred in the other groups. There was no statistical difference in the relapse rate across the groups (*p* = 0.171).

### Adverse events

There were no serious or mild adverse events.

## Discussion

Smoking is a common harmful behavior, and quitting unaided by personal willpower is challenging. Despite smokers being aware of the risks, with over 70% expressing a desire to quit, only 5% succeed (32). Acupuncture therapy has been shown to reduce the desire to smoke and attenuate the taste of tobacco (33). In this study, acupuncture and nicotine patch therapy were combined. The results showed that acupuncture combined with NRT patch intervention had a significant effect on the tobacco abstinence rate, daily cigarette consumption, expiratory CO, FTND score, and MNWS score. Due to good participant compliance in the pilot test, a 20% loss to follow-up rate was selected to calculate the sample size in the formal test, resulting in a total sample size of 88, which differed from our protocol in the preliminary test (21).

There was a significant difference in the abstinence rate at the end of treatment across the four groups, which was 63.6% in ACNP group, 40.9% in ACSNP group, 22.7% in SACNP group and 31.8% in SACSNP group, respectively. The abstinence rate in each group was consistent with previous studies (29, 34). Acupuncture combined with nicotine patches was more effective than nicotine patches or placebo patches combined with sham acupuncture, aligning with the smoking cessation results of Hyun S et al. (34) Additionally, we did not find that acupuncture therapy alone was superior to nicotine patches alone or the placebo group, consistent with the findings of Chai et al. (22) and Chae et al. (35) We also confirmed that acupuncture combined with nicotine patch therapy reduced withdrawal effects. Moreover, acupuncture for smoking cessation is safe and effective, with no adverse events occurring during the trial. The results of a meta-analysis by White AR et al. in 2014 also indicated that, although there was a lack of consistent and unbiased evidence on long-term benefits, acupuncture is effective for smoking cessation and safe when used correctly (16).

Although there was no significant difference in the change of expiratory CO across the four groups before and after treatment, the change of expiratory CO at the end of treatment in the ACNP group was significantly higher than that in the SACSNP group, indicating that acupuncture combined with nicotine therapy may significantly reduce nicotine dependence. Along with the gradual reduction in smoking amount, the CO content in expiration also gradually decreased, with most participants reaching a complete withdrawal state (35).

The FTND score of each group showed a downward trend compared with that before and after treatment, and the inter-group comparison showed that although there was no difference in the amount of change in the FTND score among the groups, the amount of change in the ACNP group was higher than others. However, there was no significant difference between the ACSNP and SACNP groups. This finding was consistent with the report by Chai et al. (22) It is worth noting that we did not select sham acupuncture points near the smoking cessation points, as reported in previous studies, but chose control points on the auricle unrelated to smoking cessation, avoiding possible bias caused by the placebo effect (36).

The results of the MNWS scale score showed that the withdrawal symptoms of participants in each group were effectively relieved after treatment. Inter-group comparison results showed that there was no difference in the change of MNWS among all groups. However, the change in the MNWS scores before and after treatment in the ACSNP and ACNP groups was significantly higher than that in the two groups containing sham acupuncture. Therefore, we concluded that the treatment groups containing true acupuncture (ACNP group and ACSNP group) had significantly better relief of withdrawal symptoms for smoking cessation than sham acupuncture combined with (sham) nicotine patch. The results of the QSU-brief score indicated no significant difference across the groups at the end of treatment, consistent with the results of Wu’s study (37). However, the change of QUS score in the ACNP and SACNP groups before and after treatment is higher than that in the other two groups, proving that the two groups containing acupuncture have a better effect in reducing smoking craving.

Acupuncture combined with nicotine patch, acupuncture alone, or pure placebo can reduce the number of cigarettes smoked. The daily cigarette consumption in the ACSNP group had a more significant reduction compared with the SACSNP group. These results suggest that acupuncture combined with nicotine patch was more effective than nicotine patch and placebo alone, which aligns with the finding from Wu et al. (29) and Hyun et al. (34) showing that combined interventions have had significantly improvement in smoking cessation compared to that of single intervention have no better efficacy.

The acupoints used in this study may differ from some previous studies because acupuncture treatment for smoking cessation has not been standardized. Standardization of acupuncture cessation is essential to enhance the credibility of clinical studies. In this study, we selected commonly used acupoints in previous studies (23, 38). During the participant recruitment phase, we informed and required each participant to receive three treatment courses per week. We established a WeChat (social media) group for patients during the treatment and reminded each participant of the booked appointment to improve treatment compliance. In addition, the acupuncture treatment regimens used in this study were safe and easy, and their combination with nicotine patches achieved better therapeutic effects than other groups.

There are some limitations in the current study, e.g., the investigation was based on self-reporting approach, which might be potentially bias, despite we tried to minimize such bias, as stated in the Materials and Methods. More objective approach will be designed to overcome such issue. Secondly, the sample size was small due to a limited budget, and we were unable to perform any subgroup analyses. There may be bias in the analysis of result indicators with large differences, such as daily smoking amount. Therefore, we recommend a multicenter, large sample randomized controlled trial with a longer follow-up time to evaluate the long-term efficacy of acupuncture and/or nicotine patches on smoking cessation. Third, we performed appropriate verbal incentives in the WeChat group chat to improve patient compliance, so the smoking cessation effect achieved in this study may not be related to acupuncture and nicotine patches alone. Finally, many confounding factors influence smoking. Although we collected data on participant smoking and smoking cessation-related factors at baseline, there was collinearity between the factors due to sample size constraints, making it impossible to clarify factors influencing smoking cessation by acupuncture. In future studies, we need to expand the sample size, explore factors associated with or modifying the effect of acupuncture for smoking cessation, and develop individualized smoking cessation interventions for each participant to improve the clinical efficacy and safety of acupuncture smoking cessation.

## Conclusion

Acupuncture combined with nicotine replacement patch therapy was more effective for smoking cessation than acupuncture alone or nicotine replacement patch alone. No adverse reactions were found in the acupuncture treatment process. A combination of acupuncture with nicotine patches was effective in terms of withdrawal rate, alleviation of tobacco dependence, relief of withdrawal symptoms, and reduction of daily smoking, making it suitable for clinical smoking cessation applications.

## Data availability statement

The original contributions presented in the study are included in the article, further inquiries can be directed to the corresponding authors.

## Ethics statement

The studies involving humans were approved by Ethical Review Committee of the Affiliated Hospital of Gansu University of Chinese Medicine. The studies were conducted in accordance with the local legislation and institutional requirements. The participants provided their written informed consent to participate in this study.

## Author contributions

RD: Data curation, Investigation, Methodology, Writing – original draft, Writing – review & editing. DR: Writing – original draft. BL: Methodology, Investigation, Writing – review & editing. YZ: Investigation, Writing – review & editing. XM: Investigation, Writing – review & editing. XZ: Investigation, Writing – review & editing. HZ: Data curation, Writing – review & editing. LZ: Data curation, Writing – review & editing. CZ: Data curation, Writing – review & editing. XJ: Investigation, Writing – review & editing. SB: Methodology, Writing – review & editing. JF: Data curation, Funding acquisition, Methodology, Writing – review & editing.

## Footnotes

^1^ http://www.chictr.org.cn/showproj.aspx?proj=61969
